# Identification, cross-taxon transferability and application of full-length cDNA SSR markers in *Phyllostachys pubescens*

**DOI:** 10.1186/2193-1801-3-486

**Published:** 2014-08-29

**Authors:** Yuan Lin, Jiang-Jie Lu, Miao-Dan Wu, Ming-Bing Zhou, Wei Fang, Yuji Ide, Ding-Qin Tang

**Affiliations:** The Nurturing Station for the State Key Laboratory of Subtropical Silviculture, Zhejiang A & F University, Zhejiang Province LinAn, 31300 P R China; Laboratory of Forest Ecosystem Studies, The University of Tokyo, 1-1-1 Yayoi, Bunkyo-ku, Tokyo, 113-8657 Japan

**Keywords:** *Phyllostachys pubescens* (*edulis*), Microsatellite (SSR), Cross-taxon transferability /polymorphism, Hybrid identification, Outcrossing-rate estimation

## Abstract

Current databases of *Phyllostachys pubescens* full-length cDNAs (FL-cDNAs) provide a rich source of sequences for the development of potential FL-cDNA simple sequence repeat (SSR) markers. We screened 10,608 *P. pubescens* cDNAs, discovering 1614 SSRs in 1382 SSR-containing FL-cDNAs. The SSRs were more abundant within transposable elements (TEs) than expressed sequence tags (ESTs) and genome survey sequences (GSSs), and specific dinucleotide repeats tended to associate with particular TE families: (TA)_n_ with *En/Spm* and (CT)_n_ with *Mutator*. A selected panel of 100 FL-cDNAs containing type I SSRs yielded 68 functional SSR markers with an average polymorphism information content (PIC) value of 0.12, among which 22 loci contained polymorphisms. These markers became less transferrable (83.1% → 69.9% → 49.3%) but more polymorphic (79.4% → 92.3% → 92.8%) with increasing phylogenetic distance (intra-genus → intra-subtribe → intra-family). Transferability and polymorphism also depended on the location of the marker, with those located in the coding region being more transferrable (69.1%) and less polymorphic (89.4%) than those in the 5′-UTR (63.4% transferable, 90.7% polymorphic) and the 3′-UTR (61.8% transferable, 91.4% polymorphic). As proof of principle, we were able to use our FL-cDNA SSR markers to identify the parental stocks in interspecific hybrids of bamboo within and beyond *P. pubescens*, and estimate the outcrossing rate for *P. pubescens*. Our research should facilitate molecular breeding in bamboo species where original genetic markers are scarce.

**Electronic supplementary material:**

The online version of this article (doi:10.1186/2193-1801-3-486) contains supplementary material, which is available to authorized users.

## Background

Bambusoideae is a subfamily of the grass family Poaceae and is further divided into nine subtribes comprising more than 80 bamboo genera and about 1400 species worldwide. Fifty genera and more than 500 species are found in China, among which *Phyllostachys pubescens* (synonym: *P. edulis*) is commercially the most important species providing the third largest source of timber and the most predominant source of bamboo shoots. *P. pubescens* plantations cover an area of 3 million ha (approximately 2% of the total forest area), which has doubled over the last 30 years and taken on a more important ecological role (Fu 2001). Compounds extracted from *P. pubescens* have recently shown potential for the treatment of obesity and other diseases (Higa et al. 2012). However, various problems associated with *P. pubescens* plantations including its simultaneous flowering intervals of more than 60 years and recovers from a limited number of clones (Janzen 1976; Watanabe et al. 1982). Additionally, the little knowledge of its basic biology, genetics and breeding system bring about the practical difficulties associated with the identification and characterization of superior genotypes.

Molecular markers developing from microsatellites, also known as simple sequence repeats (SSRs) with characterization of high genome coverage, random dispersion, co-dominant inheritance, reproducibility and amenability to automation in high throughout genotyping, have gained considerable spotlight recently. By now, microsatellite markers have been developed for several other bamboo species, e.g. six loci for *Bambusa arundinacea* (Nayak and Rout 2005), eight loci for *Sasa senanensis* (Miyazaki et al. 2009) and eight loci for *S. cernua* (Kitamura and Kawahara 2009). We identified 19 GenBank microsatellite markers in *P. pubescens* and related species (Tang et al. 2010), and 15 expressed sequence tag (EST) SSR markers for *Bambusa* species (Dong et al. 2011). Recently, the Bamboo Full-Length cDNA Project (Peng et al. 2010) has generated a vast amount of publicly-available *P. pubescens* cDNA sequence data that can be used for gene discovery, comparative genomics/transcriptomics and marker development. Microsatellites derived from cDNAs or ESTs are highly transferable to closely related species (Zhang et al. 2005) facilitating the development of gene-based maps that may increase the efficiency of marker-assisted selection through the use of candidate genes (Rossi et al. 2003; Lu et al. 2006).

Here, we report the use of *P. pubescens* full-length cDNA (FL-cDNA) sequences to 1) analyze the association between SSRs and transposable elements (TEs) in the transcriptome; 2) develop and validate FL-cDNA SSR markers and determine their transferability to other bamboo species; and 3) apply the polymorphic SSR markers to estimate outcrossing rates in *P. pubescens* and identify bamboo interspecies hybrids.

## Results and discussion

### Association between SSRs and TEs in the *P. pubescens*transcriptome

We analyzed 10,608 *P. pubescens* FL-cDNA sequences available in NCBI GenBank, representing ~7171 kb of DNA. EST-trimmer was used to remove poly(A/T) runs, and the remaining sequence data were screened using MISA, identifying 2330 SSRs in 2014 cDNAs, the remaining cDNAs lacking SSRs. The sequences were clustered with CAP3, reducing the collection to 1614 non-redundant SSRs in 1382 cDNA contigs (Additional file 1: Figure S1). Peng et al. (2010) described the distribution of SSRs in the *P. pubescens* transcriptome in detail. Therefore, we selectively analyzed the non-redundant cDNA sequences and contigs with RepeatMasker to determine the association between SSRs and TEs because previous reports have shown that many SSRs are located in TEs (Richard et al. 2008), e.g. 50% of SSRs in the human genome (Scherer 2008), and that SSRs are closely associated with TEs in rice (Akagi et al. 2001; Temnykh et al. 2001) and barley (Wei et al. 2002). The results revealed 95 TEs, representing 13.52 kb (0.27%) of the total cDNA sequence data. Further analysis showed that 29 TEs contained a total of 39 SSRs, accounting for 822 bp (6.41%) of the total TE DNA sequences. In comparison, the non-redundant EST sequence data (7089 cDNAs refined from the original 10,608 sequences) contained 1614 SSRs, accounting for 2.60% of the total cDNA sequences in length. Therefore, SSRs were approximately 2.5-, 65.4-fold more abundant in TEs compared to cDNAs (Table 1) and whole genome (0.098% based on the analysis of genome survey sequences (GSSs; Tang et al. 2010)). It is possible that SSRs within TEs are also involved in the regulation of gene expression (Tomilin 2008).

**Table 1:** **Association between FL-cDNA SSRs and transposable elements (TEs) in** ***P. pubescens***

TE family	No.	Length (bp)	No. of TE-SSR	No. of SSR-TE	TE-SSR/SSR-TE (%) (in length )	No. of SSRs with repeat units of:					
						1 nt	2 nt	3 nt	4 nt	5 nt	6 nt
**Total TEs**	95	13522	36	29	6.08	1	31	4	0	1	0
*En/Spm*	8	815	6	5	33.87	0	5	1	0	1	0
*Mutator*	28	2931	13	12	14.19	1	10	2	0	0	0
Ty1-*copia*	20	4010	1	1	0.3	0	0	1	0	0	0
Ty3-*gyspy*	17	2913	0	0	0	0	0	0	0	0	0
Other TEs	22	2853	16	11	8.70	0	16	0	0	0	0
**EST**	7089	4942281	1614	N.A.	2.60	271	489	789	30	14	21

Some studies have also suggested associations between specific SSR motifs and particular TE families, e.g. (TA)_n_ is often found in the 5′-UTR of *Micron* element transposase genes in rice (Akagi et al. 2001; Temnykh et al. 2001). We also investigated the distribution of SSRs among DNA transposons, and found they were most likely to occur in *En/Spm* elements (33.87% of the total *En/Spm* DNA sequence). Six SSRs were found in five *En/Spm* elements, with one element containing two SSRs (Table 2). *Mutator* elements were the next most likely to contain SSRs (14.19% of the total *Mutator* DNA sequence). Thirteen SSRs were found in 12 *Mutator* elements, again with one element containing two SSRs (Table 2). The situation was very different among retrotransposons, with only 0.30% of the total Ty1-*copia* DNA sequence and 0% of Ty3-*gypsy* DNA sequence made up of SSRs. More detailed investigation of specific repeat motifs showed that four of the six SSRs found in *En/Spm* elements were TA/AT repeats, and 10 of the 13 SSRs found in *Mutator* elements were CT/AG repeats. All 13 of the *Mutator* SSRs and six of the *En/Spm* SSRs were located in the 5′-UTR. It has been reported that TE molecular markers (*mPing*) showed significantly higher levels of polymorphism than all other molecular markers in closely-related rice cultivars (Monden et al. 2009). Considering that it is difficult to detect genetic variation in *P. pubescens* using ordinary markers (Lin et al. 2009; Tang et al. 2010), SSRs in TEs therefore appear to be promising markers for bamboo species.

**Table 2:** **Distribution of SSRs in** ***En/Spm*** **and** ***Mutator*** **transposons**

ID	SSR motifs	Length (bp)	Starting	Ending	Location
**SSR distribution in** ***En/Spm*** **transposons**					
FP091991	(GAGGA)_6_	30	109	138	CDS
FP091422	(TA)_22_(CA)_9_	62	12	73	5′UTR
FP097776	(TA)_23_	46	1	46	5′UTR
FP100462	(TA)_31_	62	14	75	5′UTR
FP100841	(CGG)_6_	18	38	55	5′UTR
FP100858	(AT)_29_	58	22	79	5′UTR
**SSR distribution in** ***Mutator*** **transposons**					
FP100733	(TC)_8_-(GGC)_5_	89	20	108	5′UTR
FP100664	(AG)_17_	34	32	65	5′UTR
FP094905	(CT)_17_	34	1	34	5′UTR
FP099988	(CT)_19_	38	1	38	5′UTR
FP094782	(CT)_12_	24	7	30	5′UTR
FP099842	(CT)_15_	30	4	33	5′UTR
FP091749	(CT)_23_	46	2	47	5′UTR
FP093400	(GA)_16_	32	23	54	5′UTR
FP096707	(GAA)_8_	24	36	59	5′UTR
FP096801	(TC)_8_	16	2	17	5′UTR
FP099127	(AG)_18_	36	40	75	5′UTR
FP099725	(C)_13_	13	1	13	5′UTR

### Development and polymorphism assessment of FL-cDNA SSR markers for *P. pubescens*

Original collection of 10,680 *P. pubescens* FL-cDNA sequences produced 1382 cDNA contigs containing SSRs. Sequences containing mononucleotide repeat motifs were excluded, leaving 1051 cDNA sequences containing SSRs with 2–6 nt repeats motifs (Additional file 1: Figure S1). Following the procedure already adopted for rice (Temnykh et al. 2001). We were able to design primer pairs for 583 (55%) of these cDNAs, the remainder offering either insufficient flanking DNA (over half of the SSRs were found in the 5′ or 3′ UTRs) or flanking DNA that was unsuitable for primer design. Only 325 (24.1%) of the SSRs were type I repeats (>20 bp), which offer greater potential for marker development. The 100 most promising sequences were selected for PCR validation, including dinucleotide repeats with ≥12 repeat units, trinucleotide repeats with ≥8 repeat units, tetranucleotide repeats with ≥6 repeat units, pentanucleotide and hexanucleotide repeats with ≥5 repeat units and some compound SSRs with >24 repeats (Table 3). We found that 32 of the selected cDNAs were unsuitable because the PCR failed to generate a product (four cDNAs) or generated products lacking SSRs (28 cDNAs), but the remaining 68 sequences allowed the development of FL-cDNA SSR markers (Table 3). These contained 18 compound SSRs, 19 dinucleotide repeats, 18 trinucleotide repeats, four tetranucleotide repeats, three pentanucleotide repeats and six hexanucleotide repeats. Interestingly, although 45 of the cDNAs (66.2%) generated the anticipated PCR product, 16 (23.5%) generated products with more repeats than expected, five (7.4%) generated products with fewer repeats than expected, and two (PBM050 and PBM055) generated products with different repeats and flanking sequences than those anticipated. The unanticipated amplification resulted in three SSR markers (PBM036, PBM055 and PBM 077) containing type II repeats (12–19 bp in length) and one marker (PBM079) shorter than 12 bp. In total, 67 sequences were deposited in GenBank (accession nos GU644371–GU644438). Based on BLASTX analysis, putative functions were assigned to most (66.2%) of the cDNA sequences with significant similarity to known proteins, whereas 27.9% matched unknown/hypothetical proteins and 5.9% were novel sequences (Table 3).

**Table 3:** **Characteristics of the** ***P. pubescens*** **SSR markers derived from FL-cDNAs**

No.	Marker	Accession no.		Primer sequence (5′→ 3′)	Motif	Tm (°C)	PCR fragment (bp)	PIC	Putative function
	Name	cDNA	SSR						
**1**	PBM031	FP094740	GU644371	CGCCGAGTTCCCTATTATTATTT	(AG)_6_-(AG)_7_	56	191	0	MYB-like transcription factor
				AGCACAGCCTCCGTGATTG					
**2**	PBM032	FP098085	GU644372	TTTCCCAAATAAAACCTCACC	(CCG)_7_-(CCT)_6_	56	143	0	PHD finger protein
				GTCCATTTAGGGTTCCACTGA					
**3**	PBM033	FP099510	GU644373	CTGACTGTGCGTGCGTCTC	(CG)_8_(AG)_14_	56	155	0	Small GTP-binding protein
				CTTGGTCTCGCTCATCTCCTC					
**4**	PBM034	FP098748	GU644374	TCGGCTCGGCGTGATGGAT	(GAG)_5_(GCG)_5_	62	169	0	GTP binding protein
				ATCGGCATCCGCGACTGCC					
**5**	PBM035	FP100911	GU644375	ACCGTGATGACTACCGCCGCGACC	(GTG)_7_-(GTG)_7_	62	165	0.368	U2 snRNP auxiliary factor
				TGCTGCCTCCACCCCTCCGTCC					
**6**	PBM036	FP096684	GU644376	CACATGGACCGCCTCATCC	(TA)_8_	47	169	0.259	Polypeptide-associated complex alpha subunit-like protein
				GCAACAAAACGAGAACCAGAC					
**7**	PBM037	FP101192	GU644377	TGCAAGCCTGCTATACGTTT	(TA)_7_-(TA)_6_	47	130	0	Thaumatin family protein
				GAAGTGGGAGTACATACTTCCCA					
**8**	PBM038	FP101125	GU644378	GGTCGGCTCATTTTGTAGTGT	(TC)_9_(TA)_22_	48	210	0.365	GCIP-interacting family protein-like
				CAACCTTCAGGCAATAGATTACAT					
**9**	PBM039	FP091409	GU644379	CATCCTCAGTTTCTCACCG	(TC)_12_-(CTT)_6_	53	171	0.355	Unknown protein
				CAGCTTCACCAACTTGTGG					
**10**	PBM040	FP096343	GU644380	GAATCATCTGGGAAGAAGAAGGA	(TC)_7_-(TC)_7_	51	178	0	Bicolor hypothetical protein
				TGCATTGCATTTGGCTTAGTAGT					
**11**	PBM041	FP095242	GU644381	TGGTGTTGCCTGTGACCTTAC	(TG)_8_(AG)_10_	53	167	0	typeA response regulator 1
				CCCACCTCCACCTCTACTACG					
**12**	PBM042	FP093940	GU644382	TCCTTTACGGCTTTACCCC	(GA)_7_-(AG)_6_	53	156	0.365	SAM and SH3 domain-containing protein 1
				GCCCCAGCTTAGTACACCAC					
**13**	PBM043	FP099127	GU644383	CTCACCGCCCCACCTCGCA	(AG)_13_	60	128	0	IAA15 - auxin-responsive Aux/IAA family member
				CGGCTGCTGATGCGGAGGA					
**14**	PBM044	FP095585	GU644384	AAGGCCCACGTTGCCAGAC	(AG)_20_	55	173	0.371	Bicolor hypothetical protein
				GTTCCCGTTGATGCCCCAC					
**15**	PBM045	FP098751	GU644385	TGAGCGAGGTAGTTTCATTTTAGTTA	(CA)_20_	53	132	0.322	DRE binding factor
				CCTACGACGAGTAGATTGCGAGT					
**16**	PBM046	FP094276	GU644386	CTCAGAGCAGACACTGCTTATTCC	(CT)_5_-(CT)_6_	50	102	0.395	Unknown protein
				GCGTCTTCATTGCAGCCATCT					
**17**	PBM047	FP099829	GU644387	ACCACGTTGCAGGATTCACT	(CT)_13_	53	119	0	Bicolor hypothetical protein
				CGATGAGCAGCACAACAGC					
**18**	PBM048	FP092637	GU644388	GCAAAAGAGCGCACTTGAC	(CT)_27_	53	163	0	Serine carboxypeptidase 1 precursor
				GGAGGACACTAGAGTTGGCATT					
**19**	PBM049	FP099913	GU644389	ACAGCAGATAGTCCCAAAAT	(GA)_14_	50	117	0.305	Unknown protein
				GACAGCAGGATGAAGAGCA					
**20**	PBM050	FP093015	GU644390	AGTATAGTATGTTCGTTTAAGTGG	(CA)_11_	45	137	0	Oxidoreductase
				TGTAATGTTTAAGGTTCCGT					
**21**	PBM051	FP092618	GU644391	AGACATTGTCAACTGTAAGTTGGTAGAG	(TC)_23_	50	111	0	VQ motif family protein
		(FP099842)		TTTACAAGCAATACACCCAGAAATAG					
**22**	PBM052	FP095787	GU644392	AGCGGGCAGGCTATGTATT	(TCT)_11_	51	140	0.359	ELF4-like protein
				TTGCTTCTCCCCTAATGACA					
**23**	PBM053	FP094717	GU644393	CCCCATAATCTGCTCCCTTCT	(TTC)_10_	51	102	0	KN1-type homeobox transcription factor
				GGTTCTTGGCGTATGGTATGTTC					
**24**	PBM054	FP100158	GU644394	ATCGGGAGGGATGCGGCAGC	(GGCGGA)_6_	62	121	0.305	Unknown protein
				GCGGACCAAGCGGAACACC					
**25**	PBM055	FP100601	GU644395	CATGGATGTTGTTGAGTTGAGGC	(TC)_7_	53	199	0	Nonspecific lipid-transfer protein 2 precursor
				GCACAAAGACTAGTACTCGAGGTGG					
**26**	PBM056	FP100601	GU644396	CATGGATGTTGTTGAGTTGAGGC	(CTCCAT)_6_	53	177	0	Nonspecific lipid-transfer protein 2 precursor
				GCACAAAGACTAGTACTCGAGGTGG					
**27**	PBM057	FP097951	GU644397	CGCCCACCCCTCCTTCGTCT	(ACACAG)_5_	59	111	0	Cp protein
				TCCTTGGCACGGCCACTCA					
**28**	PBM058	FP097794	GU644398	GGCCGAGATCCTCCTTTCT	(GGCGGT)_5_	59	171	0	Unknown protein
				CCATCCCCGCCTTCACCAC					
**29**	PBM059	FP094127	GU644399	ATTAGTCACGCACCGAGAAGGAA	(AGATG)_6_	55	172	0	Transcription elongation factor-related protein
				AGACGCAAGAACTCGACAGGGA					
**30**	PBM060	FP101691	GU644400	CACGCCAGCTCCAGATGCCACCAT	(CACCC)_5_	59	119	0	Sucrose transporter
				TGCCCTTCCACCTCCTCTGACCTCC					
**31**	PBM061	FP095238	GU644401	CCCTATCCCATCCTCCTCCC	(CCTCT)_5_	55	119	0	Smr domain containing protein
				GGTTGCTCACTTTCCTGCTCC					
**32**	PBM062	FP096136	GU644402	TGCTGGTTGGGTTCATCACGA	(TTCT)_7_	53	156	0	Bicolor hypothetical protein
				GAGGGTTACAACAGGGGCAAAGA					
**33**	PBM063	FP098746	GU644403	CAACGCAACGCCATTCCAAACA	(TCCA)_5_	59	138	0	U-box domain containing protein
				CACCTCCAGGCCCTGGTACTCCA					
**34**	PBM064	FP099572	GU644404	CATTTCTCATTGCCGCTGTAAC	(GAGT)_5_	53	139	0	Unknown protein
				TCCTTTGCCCTCCTCTTCCT					
**35**	PBM065	FP096965	GU644405	GTCAGTCAGGCGGCACGAG	(CG)_5_-(CGG)_9_	60	183	0	Bicolor hypothetical protein
				CGCGTAGGACGAGATCACCTC					
**36**	PBM066	FP095562	GU644406	CTCTTCACCGAAACCGAAAG	(CGG)_9_	57	137	0.477	Spliceosomal protein
				CGTTGAGGTTCCTGAGGTAGAC					
**37**	PBM067	FP098504	GU644407	GGTGCGGGTGCAGTTTATT	(CTT)_8_	51	185	0	RNA-binding protein
				AGCATCATCCGCCAGAATA					
**38**	PBM068	FP093884	GU644408	AACCGTGCACTACTTGCTCT	(TCT)_8_	51	155	0	pollenless3 mRNA
				ACCTTGTGGACGACATGGA					
**39**	PBM069	FP099427	GU644409	CCCTTTCCCTTCAACAACAA	(CCG)_8_	57	101	0.360	Alba superfamily protein
				TACCGATCCATGGCTCCTT					
**40**	PBM070	FP094239	GU644410	TCGTGCCTTTCGCCTCCTG	(TCT)_7_	55	117	0	Bicolor hypothetical protein
				CTGTACGGCCCGAACTTGTA					
**41**	PBM071	FP093953	GU644411	AGCGTCACCTCCGCCTTCT	(CGA)_8_	57	101	0	Unknown protein
				TCCTTGGCCTCGTCTTGGT					
**42**	PBM072	FP093285	GU644412	CCTCCCACTGTCACGGCACC	(CTC)_9_	59	116	0	Bicolor hypothetical protein
				GGCTGTGGCGACAAGGCTG					
**43**	PBM073	FP096973	GU644413	AGCAGCTCTACGGCAAGAAGAAG	(ATC)_8_	53	139	0	Bicolor hypothetical protein
				TGCAGCCTTGAGGAATTGAGAA					
**44**	PBM074	FP096816	GU644414	CCCACCGAAGTAATCACGC	(CTC)_8_	55	119	0	Transcription factor HBP-1a(c14)
				CTCGCACAACAAAAGAAATCA					
**45**	PBM075	FP096707	GU644415	AGTTTCCTTCTTTCCTTCCTTCCGTGGTG	(GAA)_8_	53	101	0.510	Unknown protein
				CGGCATTTGCGATTTGTGC					
**46**	PBM076	FP101632	GU644416	ATGCCTTCACCACACTTAC	(GCA)_8_	51	121	0	AP2/ERF domain protein
				CATCGTGATGTCTCCAATC					
**47**	PBM077	FP096443	GU644417	CCGCTTCCTCCCACCAAAT	(CCG)_5_	59	181	0	Bicolor hypothetical protein
				CGCAGTACAGCAGCTCCCC					
**48**	PBM078	FP095554	GU644418	CCCAAATCCAACCAGAACCA	(CGG)_11_	59	187	0	Anti-silencing protein
				GGAGGAGGCATTCGTAGGAGA					
**49**	PBM079	FP097911	GU644419	AAGGATGGTAACGTACATACA	(AT)_5_	44	159	0	Unknown protein
		(FP092888)		CATGACAAATTTAAAGGTATCA					
**50**	PBM080	FP093425	GU644420	CGAGGTTCTTGGGCTCAGTT	(AG)_13_	53	116	0.375	ATP binding protein
				ACACGCCTCCAATAAAACAAAC					
**51**	PBM081	FP097485	GU644421	TCTACTCCGTAGCCGCCTTC	(CT)_16_	56	135	0	Pyridoxamine 5-phosphate oxidase
				AGAGCCTCCATTGGATGGG					
**52**	PBM082	FP099753	GU644422	AATTTGTTGCCCTGCCTAGCT	(TC)_5_-(TC)_16_	53	148	0	Homeodomain leucine-zipper protein Hox8
				GCAAGATGAGAAGAATTAAAGCTGC					
**53**	PBM083	FP101428	GU644423	CCATTTGGCATTTGCTCCC	(GA)_15_	59	186	0	GTPase SAR1 (Sar1.1)
				GCACCCCGTAGAACCAGTCC					
**54**	PBM084	FP092513	GU644424	CTTCTCATGGGGTCAGCTACTC	(TC)_17_(AC)_16_	53	201	0.369	Brown planthopper-induced resistance protein 1 (Bi1)
				ATCACTTCTGCGATCTTGGTC					
**55**	PBM085	FP091409	GU644425	GGGGAGCCATCCTCAGTTT	(TC)_12_-(CTT)_6_	55	183	0.346	Putative precursor micro RNA R167h gene
				GCTGGCAGCTTCACCAACT					
**56**	PBM086	FP096167	GU644426	GTGGAAAATAAAGAAGCGC	(TC)_9_-(TC)_9_	51	139	0	Unknown protein
				TTCCTGCTTTTGATCTTGC					
**57**	PBM087	FP093957	GU644427	ACCCCAAGCATCCCCAAAA	(CCT)_5_-(CGC)_9_	59	166	0.373	Bicolor hypothetical protein
				CCGCAGGGAAGTCGAAGGTC					
**58**	PBM088	FP091571	GU644428	GTGTATTGGCTTTCCAGCTTTTCC	(AG)_11_	55	211	0	Knotted class 1 homeodomain protein
				TCTCCGCACGCTACTGTCCC					
**59**	PBM089	FP097920	GU644429	TCCCTTATCCACCAAACACGC	(CT)_17_	56	172	0.369	Bicolor hypothetical protein
				GCTGGCAACGACGCACCTC					
**60**	PBM090	FP097267	GU644430	AGAGTCGGATAAGGGTAGCG	(AG)_12_	53	106	0.195	Repair protein RAD23
				CGATCTCGAAGTTCGTGCC					
**61**	PBM091	FP100553	GU644431	ATAGAGGCATACAGCCGCAGAC	(AG)_14_	56	126	0.369	Macrophage migration inhibitory factor
				TAGGCACGGCATCACGGAC					
**62**	PBM092	FP099642	GU644432	GAACGCCGCATCCAGCCTCT	(TC)_13_	53	155	0	Basic/leucine zipper protein
				GGTCGGGTCCTTGGACAAAC					
**63**	PBM093	FP100738	GU644433	TCGCAGTAAACAGTCTCATCACATC	(CCT)_8_	59	150	0	Disulfide isomerase (PDIL2-2)
				TCAGGGCCACCACCTCGTCT					
**64**	PBM094	FP095169	GU644434	GATTGAGGAGCCCCAAACC	(CCG)_8_	57	257	0	DUF2372 superfamily protein
				CACAACAACCGCAAGAGCC					
**65**	PBM095	FP098630	GU644435	TTATTAGTCGAGTTTGGGTCTCC	(CCT)_8_	55	115	0.430	Unknown protein
				GGTGAACGGCATGGCTGCT					
**66**	PBM096	FP100124	GU644436	CACTCGGCTCGTCCTCGTCT	(CCTC)_6_	60	129	0	PLAC8 superfamily protein
				AGGGTGGCTAAGGCTCGTCTC					
**67**	PBM097	FP099849	GU644437	CTGCCACTCCATCCCTGCC	(CACGCG)_5_	59	101	0	Unknown protein
				CTCGATGGCGACGGCTGTT					
**68**	PBM098	FP097471	GU644438	CCCCGTCTTCTCGTCGTCT	(TCGCCG)_5_	56	169	0	BAH_BAHCC1 superfamily protein
				GACTTTGTCGGAGCCCTTGA					

One hundred and seven primer pairs finally yielded 68 FL-cDNA SSR markers for *P. pubescens*, which is towards the lower end of the 60–90% success rate previously reported in sugarcane (Cordeiro et al. 2001), barley (Thiel et al. 2003), wheat (Yu et al. 2004) and peanut (Liang et al. 2009). Squirrell et al. (2003) defined the successive loss of sequenced fragments and designed primers, until arriving at a final collection of “working SSRs” producing discrete bands of the expected size, as the “attrition rate”. Kofler et al. (2008) reported a high attrition rate when developing SSR markers from enriched libraries, BAC-end sequences and ESTs in rye, possibly reflecting the large number of TEs in the rye genome. Tero et al. (2006) found that the number of SSR markers was reduced when the markers were predominantly located within TEs. Squirrell et al. (2003) suggested that SSR marker development would be challenging in polypoid species and species such as wheat and rye with large numbers of TEs. *P. pubescens* has 2n = 48 chromosomes and is thought to be tetraploid (Li et al. 1999). The genome is >2000 Mb, which is approximately 5.4 times larger than diploid cultivated rice (Gui et al. 2007), and it contains a large number of TEs (Zhong et al. 2010; Zhou et al. 2010a, [b], [c]). The slightly higher attrition rate we encountered therefore seems reasonable when considering the chromosomal polyploidy, size and TE content of the genome*.* We also encountered a higher attrition rate in *B. oldhamii* (Li et al. 2001), a hexaploid bamboo species with a large genome (data unpublished) in which we developed 15 EST-SSR markers from 52 promising sequences selected from 3406 non-redundant ESTs (Dong et al. 2011).

We surveyed the allelic variability of the markers by genotyping 50 open-pollinated seedlings germinated from the year 2010 seedlot (Table 3). Among the 68 FL-cDNA SSR markers, only 22 (32.4%) showed polymorphism. The polymorphism information content (PIC) values of the 68 markers ranged from 0 to 0.51 with a mean value of 0.12. For the 22 polymorphic loci, the PIC values ranged from 0.19 to 0.51 with a mean value of 0.36, and the top ten markers in terms of polymorphism were PBM075, PBM069, PBM095, PBM046, PBM066, PBM080, PBm087, PBM044, PBM084 and PBM091. SSR polymorphism in *P. pubescens* is much lower than observed in cereals (Thiel et al. 2003; Yu et al. 2004), coffee (Aggarwal et al. 2007) and the rubber tree (Feng et al. 2009). Bamboo *P. pubescens* has a long flowering interval of more than 60 years (Janzen 1976; Watanabe et al. 1982). Therefore, open pollination (DNA recombination) appears to have limited the amount of replication slippage, which diversifies SSR alleles (Richards and Sutherland 1994; Jakupiak and Wells 1999). Clonal propagation in the interim periods of flowering has reduced the SSR diversity in bamboo (Nayak and Rout 2005). In a previous study, we discovered almost no allelic variation in the panel of 11 varieties and 17 provenances of *P. pubescens* using 19 GSS-SSRs (Tang et al. 2010).

### Interspecific transferability and polymorphism of *P. pubescens*FL-cDNA SSR markers

**Figure 1:**
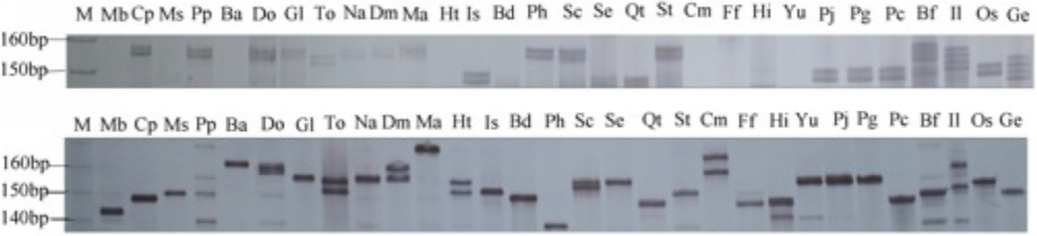
**Polyacrylamide gel electrophoresis bands representing microsatellites derived from FL-cDNA sequences, tested on a panel of selected bamboo species to evaluate transferability and polymorphism in locus of PBM042 (above) and PBM064 (nether).** M: size marker. Mb, Cp, etc.: Bamboo species abbreviations are listed in Supplementary Table 1.

Although more than 1000 bamboo species have been described, the vast majority of publically-available sequence data are derived from *P. pubescens* (Tang 2009). Therefore, the development of a set of transferable *P. pubescens* FL-cDNA SSR markers suitable for other bamboo species would help to accelerate genetic research and comparative genomics in the Bambusoideae subfamily. Previously, we developed 19 *P. pubescens* GSS-SSR markers and successfully transferred them to six other *Phyllostachys* species with an average transferability of 75.3% and 66.7% polymorphism (Tang et al. 2010). In *B. arundinacea*, 100% and 83.3% transferability were achieved with 6 SSR markers in eight other *Bambusa* species and 10 species of other genera, respectively (Nayak and Rout 2005). In *B. oldhamii*, we achieved an average 59.6% transferability and 51.4% polymorphism with 15 markers in 14 bamboo species including four species within the same genus (Dong et al. 2011). We tested the transferability and polymorphism of these 68 putative FL-cDNA SSR markers across 41 diverse species in six tribes of the Bambusoideae subfamily, as defined by Das et al. (2008) and Yang et al. (2008) (Additional file 2: Table S1 and Additional file 3: Table S2). Successful amplification became less likely with increasing phylogenetic distance from *P. pubescens*, with an 83.1% success rate within the genus *Phyllostachys*, a 79.4% success rate across genera within the subtribe Shibataeeae, and a 49.3% average success rate for other subtribes, ranging from 36.8–76.5% (Table 4 and Figure 1). In contrast, the number of markers showing polymorphism increased with phylogenetic distance, with 79.4% of markers showing polymorphism within the genus *Phyllostachys*, 91.3% showing polymorphism within the Shibataeeae, and 92.8% showing polymorphism when comparing other subtribes. Markers in coding sequences were on average the most transferrable (69.1%) and the least polymorphic (89.4%), compared to those located in 5′-UTRs (63.4% transferrable, 90.7% polymorphic) and 3′-UTRs (61.8% transferrable, 91.4% polymorphic). These trends were exacerbated with increasing phylogenetic distance. These matches the results from a metastudy of 601 loci in 35 plant species showing an average 89.8% transferability at the subgenus level, 76.4% at the genus level and 35.2% at the family level (Rossetto 2001). Interestingly, more than 17 (25%) of the markers were transferrable to more than 85% of the tested species (Additional file 3: Table S2). This success rate suggests that FL-cDNA SSRs and their flanking regions are sufficiently conserved (Zhang et al. 2005), and it is therefore possible to transfer *P. pubescens* FL-cDNA SSR markers to other bamboo species for evolutionary studies and phylogenetic reconstructions (Sharma et al. 2008).

**Table 4:** **Transferability/polymorphism of** ***P. pubescens*** **FL-cDNA-derived SSR markers across species and genera in the Bambusoideae subfamily**

Types of EST-SSR (number)	Intra-genus (***Phyllostachys***)	Inter-genus within substribe (Shibataeeae)	Inter-substribe					Average
			Melocanninae	Bambusinae	Chusqueeae	Arundinarieae	Guaduinae	
5′-UTR (41)	85.8%/81.4%	69.2%/91.3%	42.7%/93.6%	35.5%/94.4%	41.5%/88.2%	78.1%/91.4%	29.3%/90.9%	63.4%/90.7%
ORF (18)	80.6%/71.3%	70.1%/91.0%	58.3%/97.7%	57.1%/95.7%	61.1%/90.9%	75.6%/89.1%	55.6%/95.8%	69.1%/89.4%
3′-UTR (9)	75.9%/86.6%	72.2%/91.7%	41.7%/95.0%	46.0%/96.4%	22.2%/100.0%	70.9%/86.9%	33.3%/100.0%	61.8%/91.4%
Average	83.1%/79.4%	69.9%/91.3%	46.7%/94.9%	42.6%/95.0%	44.1%/90.5%	76.5%/90.2%	36.8%/93.4%

### Using polymorphic FL-cDNA SSR markers to estimate outcrossing rates and identify interspecific bamboo hybrids

Sexual propagation increases genetic diversity by creating progenies of different genotypes through recombination (i.e. outcrossing). This is advantageous for predominantly clonal plants such as most bamboo species, which rely mostly on vegetative regeneration interspersed with occasional flowering (Janzen 1976). The analysis of the reproductive system is therefore fundamental to elucidate primary genetic diversity and the structure of regenerating bamboo populations, and to adopt strategies for genetic improvement. Previous studies on the bamboo reproductive system based on field data and artificial pollination showed that self-compatibility is predominant in *Sasa* species (Nishiwaki and Konno 1990), and the selfing rate could approach 0.99 in *Merostachys riedeliana* (Guilherme and Ressel 2001). Outcrossing rate was estimated using SSR-based analysis as reported in *S. cernua* (Kitamura and Kawahara 2011).

Among the 22 polymorphic SSR markers described above, the ten most polymorphic (PIC ≥ 0.36) were used to detect polymorphisms in 50 open-pollinated half-sib seeds (year 2011) from three flowering sites in the Guangxi Province separated by at least 100 km. Polymorphism in the PBM044, PBM080 and PBM095 loci was identical in the seeds from all three flowering sites, whereas PBM084 and PBM091 featured additional alleles from Lipu, PBM069, PBM075, PBM087 and PBM091 featured additional alleles from Lingchuan, and PBM069, PBM075 and PBM084 featured additional alleles from Guanyang (Table 5). This indicated that flowering culms in different sites featured diverse SSR genotypes and produced genetically-diverse half-sib seed sources. Therefore, we used these eight polymorphic loci to estimate the outcrossing rates and other related genetic parameters for *P. pubescens* (Table 5). The overall estimates of *tm* and *ts* for three culms were 0.089 for both parameters, with no standard deviation. The estimates for individual culms showed small differences of 0.067 in Lipu and Lingchuan, and 0.133 in Guanyan, again for both parameters. Estimation of *F*_*is*_ for the overall population was 0.195, indicating homozygote excess. We found that the outcrossing rate was 0.089, estimated from eight polymorphic multilocus datasets in *P. pubescens*, which is slight lower than the 0.148 reported in *S. cernua* using six multilocus SSR datasets (Kitamura and Kawahara 2011). This indicated that the reproductive system of *P. pubescens* predominantly involves self-fertilization with an adequate proportion of crossing to ensure genetic diversity as reported for *S. cernua* (Kitamura and Kawahara 2011).

**Table 5:** **Seed number, estimated outcrossing rates and relative parameters for each of three** ***P. pubescens*** **flowering culms at 8 loci**

Flowering site (county)	N	***tm***	***ts***	***Fis***	Genotype							
					PBM044	PBM069	PBM075	PBM080	PBM084	PBM087	PBM091	PBM095
Lipu	50	0.067 (0.0)	0.066 (0.0)	0.182	p			p	p		p	p
Lingchuan	50	0.067 (0.0)	0.067 (0.0)	0.173	p	p	p	p		p	p	p
Guanyan	50	0.133 (0.0)	0.135 (0.0)	0.231	p	p	p	p	p			p
Average		0.089 (0.0)	0.089 (0.0)	0.195								

N the number of analyzed seeds; tm multi-locus outcrossing rate and standard error in parentheses; *ts*, single-locus outcrossing rate and standard error in parentheses. Fis inbreeding coefficient; p polymorphism.

**Figure 2:**
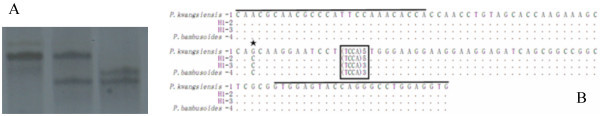
**A, Microsatellite DNA fingerprints of** ***P. kwangsiensis*** **(line 1),** ***P. bambusoides*** **(line 3) and a presumed hybrid (line 2) at locus PBM063. B**, Alignment of the nucleotide sequences of the microsatellite alleles at locus PBM014 amplified from *P. kwangsiensis*, *P. bambusoides* and two presumed hybrids. Nucleotides conserved among these sequences (relative to *P. kwangsiensis*) are shown by dots. The lines indicate the primer sequences used to amplify this microsatellite locus. The box highlights the microsatellite. The suffix numbers after bamboo species correspond to the DNA bands marked in part (a).

**Figure 3:**
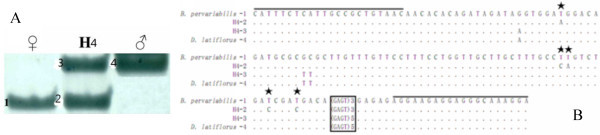
**A, Microsatellite DNA fingerprints of** ***Bambusa pervariabilis*** **,** ***Dendrocalamus latiflorus*** **and their presumed hybrids at locus PBM064. B**, Alignment of the nucleotide sequences of the microsatellite alleles at locus PBM064 amplified from *B. pervariabilis*, *D. latiflorus* and their presumed hybrids. Nucleotides conserved among these sequences (relative to *B. pervariabilis*) are shown by dots. The lines indicate the primer sequences used to amplify this microsatellite locus. The box highlights the microsatellite. The suffix numbers after the bamboo species correspond to the DNA bands marked in part (A).

The grow-out test for bamboo interspecific hybrids is time-consuming and laborious because it involves growing plants to maturity (which takes at least 5 years), assessing several anatomical, morphological and floral (long-term interval) characteristics that distinguish the hybrid. The polymorphic SSR markers could also help in the rapid and accurate identification of interspecies hybrids, as reported in poplar (Rajora and Rahman 2003) and wheat-barley (Malysheva et al. 2003). To obtain proof of principle that our novel SSR markers are suitable for hybrid characterization, we next selected several highly-transferable and polymorphic FL-cDNA SSR markers. PBM032, PMB049, PMB063 and PMB064, each with a number of species-restricted alleles, were used to test uncharacterized bamboo samples. Marker PMB063 identified the parental species in one hybrid as *P. kwangsiensis* and *P. bambusoides*, because all sequenced bands contained the (TCCA)_n_ motif although with a variable number of repeats (Figure 2). Similarly, marker PMB064 identified the parental species *B. pervariabilis* and *Dendrocalamus latiflorus* which are distantly related to *P. pubescens*, with a variable number of repeats in the (GAGT)_n_ motif (Figure 3). As previously shown using GSS-SSR markers, such high levels of transferability and polymorphism within the Bambusoideae subfamily should allow the use of FL-cDNA SSR markers to identify interspecific hybrids and their parents, both within the genus *Phyllostachys* (Tang et al. 2010) and in more distant taxa within subtribe of Shibataeeae (Lu et al. 2009). We have also developed several putative EST-SSR markers in *B. oldhamii* and have used these to identify some other sympodial bamboo interspecies hybrids (Wu et al. 2009; Dong et al. 2011). The SSR markers developed in the present study were used to identify not only interspecific hybrids from monopodial *Phyllostachys* but also intergeneric hybrids with sympodial rhizomes, which are distantly related to *P. pubescens*. Our data confirmed that microsatellites, especially SSR markers based on cDNAs and ESTs, are ideal for the identification of bamboo interspecies hybrids.

## Conclusions

Our data provide insight into the association between SSRs and TEs in FL-cDNAs from the *P. pubescens* transcriptome, allowing us to develop and evaluate 68 FL-cDNA SSR markers that can be used in *P. pubescens* and partially for many other bamboo species, to estimate the reproductive system of *P. pubescens* and identify several interspecific hybrids. These FL-cDNA SSR markers enrich the molecular marker resources currently available for bamboo. When a large set of polymorphic markers becomes available, we can use genome-wide association mapping in bamboo, in the absence of structured populations, to identify markers for traits of interest that can be used for marker-assisted selection in the Bambusoideae subfamily.

## Methods

### Full-length cDNA mining and SSR/TE detection

We obtained 10,608 FL-cDNA sequences from NCBI Entrez (http://www.ncbi.nlm.nih.gov/) on July 1, 2010. These cDNA sequences were assembled from five cDNA libraries constructed from breaking-out shoots, young (40-cm) shoots and young leaves from plants, and shoots and roots from germinated seeds (Peng et al. 2010). We used EST Trimmer (http://pgrc.ipk-gatersleben.de/misa/download/est_trimmer.pl) to remove poly(A/T) runs from the 5′ and 3′ ends until there were no occurrences of (T)_5_ or (A)_5_ within a 50-bp range. Redundant sequences were eliminated and overlapping sequences were spliced together using CAP3 (http://seq.cs.iastate.edu/cap3.html) (Huang and Madan 1999).

After pre-treatment, we used MISA (http://pgrc.ipk-gatersleben.de/misa/misa.html) to screen for SSRs including mononucleotide repeats ≥10 bp in length, dinucleotide to hexanucleotide repeats with ≥6 repeat units, and interrupted composite SSRs with ≤100 bp of intervening DNA. Putative annotations were assigned to non-redundant ESTs containing SSRs using BLAST against the Moso Bamboo cDNA Database (http://202.127.18.228/mbcd/) and the Gramene Ontologies Database (http://archive.gramene.org/plant_ontology/). TEs were identified using RepeatMasker and RepeatProteinMask (http://www.repeatmasker.org) based on similar elements present in the rice genome, and SSRs within TEs were screened using MISA with the same parameters as above. Additional file 1: Figure S1 provides a flow chart for the data mining and marker development process.

### Plant material and DNA extraction

We used *P. pubescens* samples collected from the Anji Bamboo Germplasm Garden, Anji, Zhejiang Province, to identify and characterize putative FL-cDNA SSR markers. The polymorphism of these SSR markers was evaluated using 50 seedlings germinated from an open-pollinated seedlots (mixed seed sources, mainly from different flowering sites in the counties of Lipu, Lingchuan and Guanyang, Guangxi Province in the year 2010). Another 50 seedlings were germinated from open-pollinated half-sib seeds (year 2011) from three flowering culms in the same three counties (>100 km between sites) and were used to estimate the *P. pubescens* outcrossing rate. We obtained 41 representative bamboo species from 38 genera within six subtribes mainly found in China to test the transferability and polymorphism of the FL-cDNA SSR markers (Additional file 2: Table S1). We obtained three *Phyllostachys* interspecific hybrids from Jiangxi Province, China, and two intergeneric hybrids from Yoshinaka Bamboo Germplasm Garden, Fukuoka, Japan, for the hybrid identification tests. Genomic DNA was extracted from young leaves using the hexadecyltrimethylammonium bromide (CTAB) method (Doyle and Doyle 1987), with some modifications.

### Amplification and sequencing of SSR loci

Primer pairs designed according to the available cDNA sequences were synthesized by Shanghai Sangon Biological Engineering Technology & Services Co., Ltd. *P. pubescens* DNA was amplified in 20-μl reactions comprising 50–100 ng of template DNA, 0.2 μM of each primer, 200 μM of each dNTP and 1 unit of *Taq* DNA polymerase with 1× PCR universal buffer (10 μM Tris–HCl, pH 8.3 at 25°C; 50 μM KCl), and 1.5 μM MgCl_2_ (Shanghai Sangon Biological Engineering Technology & Services Co., Ltd). The reaction was heated to 95°C for 5 min using an ABI PE9700 thermocycler, followed by 30 cycles of 1 min denaturation at 95°C, 1 min annealing at 46–59°C depending on the primer pair (Table 3), and 2 min extension at 72°C, followed by a final hold at 72°C for 5 min. Amplified microsatellite loci were tested in 41 diverse species in six tribes of the Bambusoideae subfamily (Table 4) and interspecific hybrids (Figures 2 and 3). The annealing temperature was lowered by 2–5°C according to the evolutionary distance between species based on molecular markers (Das et al. 2008) and nuclear and chloroplast sequences (Yang et al. 2008), as suggested by Rossetto (2001). PCR products were separated on 6% polyacrylamide denaturing gels, and marker bands were revealed by silver staining as described by Panaud et al. (1996). Specific bands were excised directly from the silver staining polyacrylamide gel, purified using the EZ-10 Spin Column DNA Gel Extraction Kit (Biobasic Inc.) and ligated into the pUC18 vector (TaKaRa, Japan). Three positive clones for each bamboo species were selected for sequencing using BigDye terminator V3.1 in a cycle sequencing protocol according to the manufacturer’s specifications (PE Applied Biosystems, ABI PRISM 3100-Avant Automatic DNA Sequencer). Vector sequences were removed then edited using Vector NTI software (version 10.0, Invitrogen Co., USA). Sequences were deposited in NCBI GenBank (accession nos GU644371–GU644438).

### Data analysis

The polymorphism information content (PIC) (Botstein et al. 1980) of our SSR markers was determined using Powermarker v3.25 (Liu and Muse 2005). All 68 selected primer pairs were used to amplify template DNA from 41 bamboo species covering 35 genera in six subtribes (Additional file 2: Table S1) and the statistical methods of Nayak and Rout (2005) and Sharma et al. (2009) were used to calculate the cross-taxon transferability and polymorphism (Additional file 3: Table S2), in which polymorphism is calculated only from the loci that were successfully transferred across taxa (Rossetto 2001). Single locus and multilocus outcrossing rates and relative parameters were analyzed separately under the mixed mating model of Ritland & Jain (1981) and Ritland (2002), implemented using MLTR v3.4 (Ritland 1996).

## Electronic supplementary material

Additional file 1: Figure S1: Scheme used for database mining and the development of SSR markers from *P. pubescens* FL-cDNA sequences. (DOC 31 KB)

Additional file 2: Table S1: Species used to test cross species/genus amplification of *P. pubescens* FL-cDNA SSR loci. (XLS 34 KB)

Additional file 3: Table S2: Cross species/genus amplification of *P. pubescens* FL-cDNA SSR loci. (XLS 87 KB)
