# Supplementary material for: Identification, cross-taxon transferability and application of full-length cDNA SSR markers in Phyllostachys pubescens
Source: Springerplus. 2014 Aug 29;3:486. doi: 10.1186/2193-1801-3-486 (PMC4161718; doi:10.1186/2193-1801-3-486)
Supplement: Supplementary file 1 — Additional file 1: Figure S1: Scheme used for database mining and the development of SSR markers from P. pubescens FL-cDNA sequences. (DOC 31 KB) [file 40064_2014_1193_MOESM1_ESM.doc]

Remove Poly(A/T) with EST-trimmer

SSR exploring with MISA search

Cluster analysis with CAP3

Primers designed using Primer Premier 5.0

**Supplementary Figure 1 Scheme used for database mining and development of genetic SSR markers from *Phyllostachys pubescens* full-length cDNA sequences.**
